# From Data to Decisions: Harnessing Multi-Agent Systems for Safer, Smarter, and More Personalized Perioperative Care

**DOI:** 10.3390/jpm15110540

**Published:** 2025-11-06

**Authors:** Jamie Kim, Briana Lui, Peter A. Goldstein, John E. Rubin, Robert S. White, Rohan Jotwani

**Affiliations:** 1 Weill Cornell Medical, Cornell University, New York, NY 10065, USA; 2 Department of Anesthesiology, Weill Cornell Medicine, Cornell University, New York, NY 10065, USA; brl4005@nyp.org (B.L.); pag2014@med.cornell.edu (P.A.G.);; 3 Department of Medicine, Weill Cornell Medicine, Cornell University, New York, NY 10065, USA; 4 Feil Family Brain and Mind Research Institute, Weill Cornell Medicine, Cornell University, New York, NY 10065, USA

**Keywords:** artificial intelligence, machine learning, anesthesiology, perioperative care, patient safety, personalized medicine, predictive modelling, risk stratification

## Abstract

**Background/Objectives**: Artificial intelligence (AI) is increasingly applied across the perioperative continuum, with potential benefits in efficiency, personalization, and patient safety. Unfortunately, most such tools are developed in isolation, limiting their clinical utility. Multi-Agent Systems for Healthcare (MASH), in which autonomous AI agents coordinate tasks across multiple domains, may provide the necessary framework for integrated perioperative care. This critical review synthesizes current AI applications in anesthesiology and considers their integration within a MASH architecture. This is the first review to advance MASH as a conceptual and practical framework for anesthesiology, uniquely contributing to the AI discourse by proposing its potential to unify isolated innovations into adaptive and collaborative systems. **Methods**: A critical review was conducted using PubMed and Google Search to identify peer-reviewed studies published between 2015 and 2025. The search strategy combined controlled vocabulary and free-text terms for AI, anesthesiology, perioperative care, critical care, and pain management. Results were filtered for randomized controlled trials and clinical trials. Data were extracted and organized by perioperative phase. **Results**: The 16 studies (6 from database search, 10 from prior work) included in this review demonstrated AI applications across the perioperative timeline. Preoperatively, predictive models such as POTTER improved surgical risk stratification. Intraoperative trials evaluated systems like SmartPilot and Navigator, enhancing anesthetic dosing and physiologic stability. In critical care, algorithms including NAVOY Sepsis and VentAI supported early detection of sepsis and optimized ventilatory management. In pain medicine, AI assisted with opioid risk assessment and individualized pain-control regimens. While these trials demonstrated clinical utility, most applications remain domain-specific and unconnected from one another. **Conclusions**: AI has broad potential to improve perioperative care, but its impact depends on coordinated deployment. MASH offers a unifying framework to integrate diverse agents into adaptive networks, enabling more personalized anesthetic care that is safer and more efficient.

## 1. Introduction

The origins of a “thinking machine” can be traced back to the early nineteenth century, when Charles Babbage designed his “difference engine,” a mechanical calculator intended to solve mathematical equations [1]. A century later, Karel Čapek’s 1920 play *R.U.R. (Rossum’s Universal Robots)* introduced the word “robot” into the public imagination, reflecting a cultural fascination with the idea of artificial people [2]. By 1950, Alan Turing posed the seminal question, “Can machines think?” [3], and in 1956, John McCarthy coined the term “artificial intelligence” (AI) at the Dartmouth Summer Research Project on Artificial Intelligence [4]. Since then, AI has developed from theoretical speculation into practical technology, becoming simultaneously disruptive and transformative across nearly every sector of society.

In medicine, and anesthesiology in particular, AI applications are diverse and expanding. Proposed uses include patient blood management [5], difficult airway prediction [6], identification of patients at risk for readmission after intensive care unit (ICU) discharge [7], and remote physiologic monitoring [8]. Anticipated benefits from widespread adoption include cost reduction, expanded access to care, improved efficiency, and reduced clinician burnout [9,10,11]. More fundamentally, AI holds the promise of enhancing clinical decision-making, thereby improving patient safety and outcomes [12].

At its core, AI encompasses multiple computational approaches. Machine learning (ML) uses algorithms trained on historical data to make predictions without explicit programming. Deep learning, a subset of ML, leverages neural networks to analyze high-dimensional data such as physiologic waveforms and imaging with superior predictive accuracy [13]. Natural language processing (NLP) extracts and structures information from unstructured text such as clinical notes, while large language models (LLMs) extend these capabilities to generate human-like text, supporting clinical documentation and patient communication [13]. Collectively, these technologies are increasingly being applied in perioperative care, from individualized risk prediction to automation of routine tasks.

Recent work [14] has advanced the concept of Multi-Agent Systems for Healthcare (MASH), in which multiple specialized AI agents collaborate in a distributed but coordinated manner to optimize patient care across the healthcare continuum. This approach is particularly relevant for anesthesiology, where clinicians manage high-acuity patients across multiple care phases—preoperative evaluation, intraoperative management, postoperative recovery, and extensions into critical care and pain medicine. Perioperative anesthesiology lies at the center of surgical care, encompassing high-risk, high-volume patient management across multiple care transitions. The dynamic nature of this environment makes it particularly suited for and necessitates data-driven, systems-based approaches to such patient care. Applying the MASH framework to anesthesiology represents a novel conceptual advancement, as it reframes isolated AI tools into a unified interoperable network capable of real-time adaptation and decision support across the perioperative continuum.

This critical review aims to synthesize current applications of AI in perioperative anesthesiology and to explore how these tools may be integrated into a MASH-based framework. By examining existing evidence across the perioperative timeline, we seek to highlight opportunities, challenges, and future directions for building a coordinated AI ecosystem to support anesthesiologists and advance personalized medicine in perioperative care.

## 2. Materials and Methods

The critical review followed the methodological guidelines outlined by Popay et al. for conducting systematic narrative reviews [15]. The objective of this focused review was to examine the application of AI and MASH frameworks in perioperative care, with a particular focus on their role in preoperative risk stratification, intraoperative decision support, postoperative recovery, and integration across the continuum of anesthesiology practice. A secondary aim was to assess how emerging AI tools can be synthesized into coordinated systems to improve patient safety, workflow efficiency, and personalized care.

A literature search was conducted using PubMed and Google Scholar. For both databases, search terms included combinations of “artificial intelligence”, “machine learning”, “multi-agent systems”, “perioperative care”, “anesthesiology”, “critical care”, and “pain management”. The search strategy focused on studies published between January 2015 and June 2025, reflecting the period of rapid advancement in AI applications within perioperative and anesthesiology settings.

Eligible studies included randomized controlled trials (RCTs) and clinical trials that reported on AI or multi-agent frameworks in perioperative, anesthesia, or critical care settings. Conference proceedings, editorials, and opinion pieces were excluded unless they provided unique insights into conceptual frameworks or implementation strategies. Only articles published in English and involving human subjects were considered. In addition to the database search, eight studies were manually added based on prior research within the author group. These studies did not appear in the automated search results despite meeting eligibility criteria and were included to ensure comprehensive coverage of conceptually foundational work relevant to the evolution of AI and systems-based approaches in healthcare.

Data extraction was performed by the authors to capture study design, population, interventions or AI applications, outcomes assessed, and relevance to perioperative or anesthesiology practice. Screening proceeded in two stages. Two reviewers (J.K. and B.L.) independently screened titles and abstracts, followed by a full-text review of eligible articles. Discrepancies were resolved by discussion, with adjudication by a third reviewer (J.R. or R.J.) as needed.

The narrative synthesis was guided by Popay et al.’s framework [15], which emphasizes textual description, exploration of relationships within and across studies, and development of a conceptual model to integrate diverse findings. Findings are organized along the perioperative timeline (preoperative, intraoperative, postoperative, and critical care/pain management domains), with cross-cutting themes such as ethics, interoperability, and system-level integration highlighted.

## 3. Results

**Figure 1:**
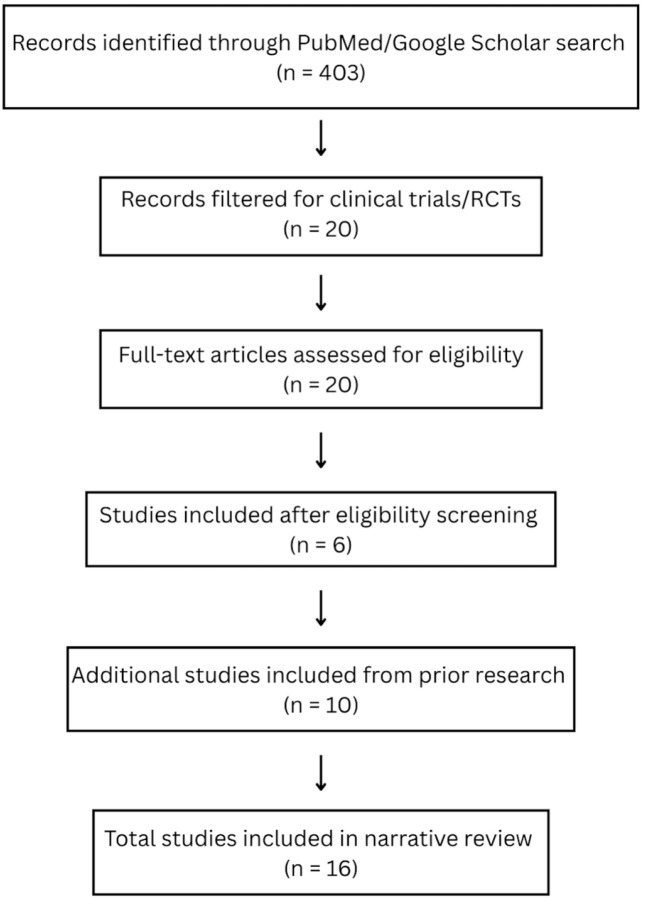
PRISMA flow diagram of study. This diagram illustrates the flow of information through the different phases of the systematic review. A total of 403 records were identified through database searching, and an additional 10 records were identified through other sources. After removing duplicates and filtering for clinical trials/RCTs, 20 records remained for screening. Ultimately, 16 studies met the inclusion criteria and were included in the qualitative and/or quantitative synthesis.

The search strategy yielded 20 studies, but ultimately, 6 studies demonstrated AI applications across the perioperative timeline [Figure 1]. Ten additional studies were added by authors R.J. and J.R. based on prior research work. These works demonstrate AI agents with utility and usefulness in perioperative medicine, but did not appear in any direct search term related to anesthesiology or anesthesiology subspecialties. A total of 16 studies were discussed for their work on specific trials related to the use of AI across the perioperative timeline. Preoperatively, predictive models could improve surgical risk stratification, while intraoperative trials evaluated systems that could enhance anesthetic dosing and physiologic stability. In critical care, algorithms assisted with early sepsis detection, and in pain medicine, AI assisted with opioid dependence risk assessment. However, while these trials demonstrate the benefits of AI in clinical practice, most applications remain domain-specific and disconnected.

### 3.1. Preoperative Applications

#### 3.1.1. Database Studies

AI has shown growing promise in preoperative risk prediction and patient optimization. Eyth et al. (2025) developed and validated the TRANSFUSE risk model, which integrates 24 preoperative variables, including American Society of Anesthesiologists physical status, international normalized ratio, surgical complexity, and liver disease, to predict intraoperative transfusion requirements [16]. With an area under the curve (AUC) of 0.93, the model offers a practical tool for surgical teams to anticipate transfusion needs while minimizing unnecessary preparation. In an investigation focusing on the patient experience before surgery, Yahagi et al. (2024) tested the feasibility of a ChatGPT-based intervention (ChatGPT-3.5, OpenAI) to reduce preoperative anxiety [17]. Patients randomized to conversational AI counseling significantly reduced preoperative anxiety compared with the control group, but no overall difference in the Japanese State–Trait Anxiety Inventory (STAI) self-report questionnaire scores was observed.

#### 3.1.2. Additional Studies

A recent review by Yoon et al. highlights the growing use of AI-based predictive models that analyze data like vital signs, comorbidities, and lab results to identify high-risk patients and to guide preoperative interventions, such as medical workup, specialist referrals, or optimization of conditions like glycemic control, to reduce surgical complications [18]. Other AI-based calculators, such as POTTER (Predictive Optimal Trees in Emergency Surgery Risk) [19], integrate patient demographics, comorbidities, laboratory values, imaging data, and surgical history to estimate procedural risk and recommend tailored optimization strategies for both surgeons and anesthesiologists alike.

Overall, AI is playing an expanding role in enhancing preoperative care and shared decision-making among patients, anesthesiologists, and surgeons. By analyzing clinical and demographic data, AI tools can not only identify high-risk patients and guide targeted interventions to reduce complications, but also improve patients’ experience before surgery. This highlights the potential of AI to act as adjuncts to traditional preoperative workflows and underscores the need for iterative refinement of these tools.

### 3.2. Intraoperative Applications

#### 3.2.1. Database Studies

The intraoperative setting has been the most fertile ground for AI adoption, with systems designed to optimize hemodynamic stability and anesthetic management. Zaouter et al. (2016) evaluated the feasibility of a fully automated closed-loop intravenous anesthesia delivery robot, named McSleepy, for patients undergoing cardiopulmonary bypass in cardiac surgeries [20]. In this pilot study, the system successfully maintained anesthesia without manual override in 80% of the 20 enrolled patients by continuously monitoring BIS for hypnosis, Nociception Index for analgesia, and Train-of-Four counts for adequate neuromuscular blockade and adjusted infusion rates in real time without clinician input.

Several other studies assessed AI for improving intraoperative analgesia and hemodynamic control. Fuica et al. (2023) demonstrated that nociception-guided fentanyl dosing using the NOL™ index (PMD-200, Medasense Biometrics Ltd., Ramat Gan, Israel), an AI-derived multiparameter monitor, significantly reduced postoperative pain scores compared to conventional hemodynamic-guided dosing [21]. Sribar et al. (2023) investigated the Hypotension Prediction Index (HPI), a novel machine learning-guided algorithm that can predict hypotensive events using high-fidelity analysis of pulse-wave contour, during thoracic surgery, and found that patients in the HPI group experienced significantly fewer and shorter hypotensive episodes compared with standard pulse contour-guided therapy [22].

Machine learning has also been applied to predict intraoperative recovery trajectories. Skitek et al. (2024) compared spinal and general anesthesia for endourological surgery while applying neural networks and regression models to predict recovery outcomes [23]. Their machine learning models reliably estimated postoperative ambulation times and recovery durations, demonstrating the feasibility of AI in optimizing perioperative workflows.

#### 3.2.2. Additional Studies

Furthermore, AI-driven systems like SmartPilot [24] View (Drägerwerk AG, Lübeck, Germany) and Navigator(GE Healthcare, Helsinki, Finland) [25] provide real-time guidance on anesthetic dosing by automatically combining drug pharmacodynamics with physiologic monitoring. These tools support anesthesiologists in maintaining adequate anesthetic depth while minimizing hemodynamic instability and avoiding excessive sedation. By continuously analyzing intraoperative patterns such as heart rate variability, blood pressure trends, and ECG features, they can offer early warnings for impending physiologic deterioration and propose corrective measures. A comprehensive review of such advances in AI-guided anesthetic delivery by Cai et al. (2025) indicates that patients managed with SmartPilot had better intraoperative outcomes, such as fewer instances of severe hypo- and hypertension, shorter times to tracheal extubation, and reduced early postoperative pain scores, compared to those managed using traditional anesthesia monitoring methods [26].

By leveraging real-time physiologic data and predictive modeling, AI systems can optimize drug delivery, anticipate adverse events, and support clinical decision-making without requiring constant clinician input. These technologies have been associated with improved patient outcomes, including greater hemodynamic stability, reduced postoperative pain, and more efficient recovery trajectories, highlighting AI’s growing role in advancing intraoperative care.

### 3.3. Critical Care and Pain Medicine Applications

#### Additional Studies

AI is playing an increasingly pivotal role in critical care and pain medicine. In the ICU, AI has been leveraged for early detection and dynamic treatment strategies. Tools like the NAVOY Sepsis algorithm and other computerized clinical decision support systems leverage real-time electronic health record data like vital signs, laboratory values, and clinical documentation to identify patients at risk for sepsis earlier than traditional methods, allowing for more timely intervention and potentially reducing morbidity and mortality [27,28]. Similarly, VentAI, a reinforcement learning-based algorithm, dynamically models and suggests optimized mechanical ventilation strategies for ICU patients by predicting how various ventilator settings will impact patient outcomes, thus promoting lung-protective ventilation and improving recovery [29].

In parallel, AI applications in pain medicine are emerging to support more personalized care: machine learning models analyze patient histories, pain profiles, treatment responses, and psychosocial factors to tailor analgesic regimens with greater precision. In fact, an AI-based screening tool, which was trained on a reference dataset from structured interviews conducted by clinical staff who manually screened more than 50,000 patients using the Drug Abuse Screening Test, was found to be at least as effective as usual care in facilitating addiction medicine consultations, led to a significant reduction in 30-day readmissions, and proved to be cost effective in improving opioid use disorder care [30]. The AI screener, which was a convolutional neural network embedded in the EHR, analyzed clinical notes in real time and issued alerts prompting addiction medicine consultation. In a pre–post study, it maintained consultation rates (1.35% vs. 1.51%) while significantly reducing 30-day readmissions (14% to 8%, OR 0.53, 95% CI 0.30–0.91) at a cost of nearly $6800 per readmission avoided. Rather than relying on simple keywords, the model was able to leverage concept-level patterns in clinical documentation to identify risk for opioid use disorder.

Moreover, predictive algorithms are being developed to identify individuals at elevated risk for opioid use disorder by incorporating data on prescription patterns, comorbid mental health conditions, and social determinants of health [31]. These advancements reflect the growing integration of AI into high-stakes clinical settings, where early recognition and individualized treatment plans are essential for improving patient outcomes and minimizing harm.

Overall, AI has been transforming critical care and pain medicine by enabling earlier detection, personalized treatment, and more efficient care delivery. In the ICU, AI-driven tools enhance early identification of conditions like sepsis and optimize interventions such as mechanical ventilation, while in pain management, machine learning models support tailored analgesic plans and improve opioid use disorder screening and outcomes. Together, these innovations demonstrate AI’s growing capacity to improve precision and safety in complex clinical environments.

### 3.4. Non-Operating Room Anesthesia (NORA)

#### Additional Studies

AI in NORA has also improved procedural sedation and airway management. A recent review by Pardo et al. delineates the various ways in which AI enhances care in non-operating room anesthesia settings [32]. AI-driven diagnostic systems were found to help with the timing of sedation, with a computer-aided diagnosis system being developed to identify the remaining parts to be examined in real-time endoscopic procedures to help anesthesiologists adjust the appropriate degree of sedation. Such technology was found to lead to shorter emergence and recovery times as well as higher satisfaction among patients, without impacting total propofol dosage or vital signs. Another valuable feature described was a new machine learning algorithm that used clinical features of the Mallampati score, age, and sternomental distance, as well as other variables such as sex, height, weight, body mass index, and neck circumference, to generate a more precise prediction of patients with potentially difficult airways [32]. Therefore, while much of AI integration has centered on the operating room, these findings underscore the growing role of AI in supporting anesthesiologists in NORA settings.

### 3.5. Emerging Frameworks: Multi-Agent Systems for Healthcare (MASH)

**Figure 2:**
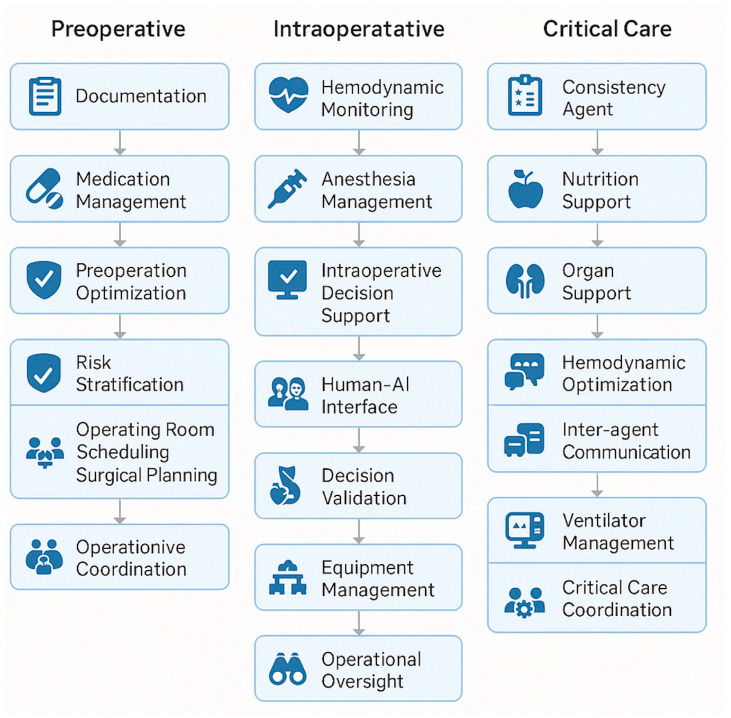
This diagram depicts an example of a modular architecture of a Multi-Agent System for Healthcare (MASH), composed of specialized artificial intelligence agents assigned to discrete perioperative and critical care functions. The framework is organized across three major phases of anesthesiology care—preoperative, intraoperative, and critical care—each containing agents responsible for domain-specific tasks in medical optimization, clinical decision support, and administrative functions. Arrows indicate a hypothetical flow of information and decision-making between agents, illustrating how modular AI entities coordinate to support continuity of care and operational integration across the perioperative continuum.

Moritz et al. introduced MASH, in which autonomous AI “agents” collaborate to optimize patient care across the perioperative timeline [14]. Each agent specializes in tasks such as data interpretation, workflow optimization, or patient monitoring, but communicates with others in real time. We propose extending MASH specifically to anesthesiology, enabling AI coordination across preoperative, intraoperative, and postoperative domains. To illustrate how this network might function in clinical practice with greater depth, Figure 2 depict several examples of agent interactions. For instance, a preoperative agent could assess a patient’s reported penicillin allergy, determine it to be low-risk, and recommend cefazolin with a test-dose protocol; it would then relay this information to the intraoperative agent, which would monitor for adverse reactions in real time and guide antibiotic administration accordingly [Figure 2]. Intraoperative events such as unexpected blood loss or hypotension could similarly be communicated forward to postoperative agents, automatically informing care planning decisions such as postoperative monitoring intensity, discharge timing, or handoffs to critical care [Figure 2]. In ICU settings, MASH agents can compound and cross-reference data across domains, such as ventilator parameters, hemodynamics, and sepsis indicators, to improve detection and intervention timing [Figure 2].

Expanding upon these interactions, a preoperative agent could analyze patient data to identify individuals at high risk for developing chronic postsurgical pain based on prior opioid use, psychological comorbidities, or surgical factors. This agent could then collaborate with an administrative scheduling agent to arrange a preoperative consultation with a pain specialist. Based on those recommendations, the intraoperative agent could be prompted to adjust the anesthetic plan, for example, by recommending regional anesthesia, specific adjuvants, or multimodal analgesia strategies. This information would be relayed to postoperative agents, which could interpret early postoperative pain scores and vital signs to trigger low-threshold alerts for rescue analgesia, consider placing nerve catheters, or escalate pain management plans. Finally, a discharge coordination agent could synthesize inpatient interventions and pain trajectories to guide outpatient follow-up, prioritizing high-risk patients for early evaluation and supporting continuity of care through structured feedback to primary or specialty care providers.

This coordinated flow of information transforms perioperative care from a series of isolated decisions into an integrated, responsive network that adapts in real time to patient needs. By leveraging MASH architecture, the entire perioperative continuum can function as a cohesive system. As previously demonstrated by Churpek et al., linking early warning signals across phases of care has been associated with improved patient outcomes, and MASH models provide the technological infrastructure to enable this type of system-wide situational awareness and learning [33].

## 4. Discussion

Our review highlights the rapid expansion of AI across the perioperative continuum. Preoperative applications emphasize predictive analytics for surgical risk and optimization; intraoperative tools enhance anesthetic delivery and early detection of physiologic instability; critical care applications leverage reinforcement learning and sepsis prediction; and pain medicine tools are emerging to address potential opioid use disorders. Despite their promise, these tools have largely been developed in isolation from each other, limiting their ability to impact patient care holistically. This is likely because the work of developing AI agents or algorithms in perioperative medicine has been occurring for at least a decade, but likely longer in various healthcare environments. Given the computational resources and research logistics/computing power required to build coordinated systems, it has historically been most feasible to develop individual agents with limited, but specific datasets for both testing and validation purposes, rather than build one single complete perioperative AI model across all domains. MASH represents a novel framework to coordinate these diverse AI functions, offering a pathway to seamless, system-wide integration in anesthesiology.

The authors recognize the limitations and complexity of searching for all potential AI algorithms that might be useful in creating a hypothetical MASH system. In this review, we highlight that our search identified six agents, yet our overall framework included an additional eight agents. Given the complexity of perioperative care and how many medical divisions anesthesiologists collaborate with for patient care (ie, general medicine, intensivists, surgeons, emergency medicine specialists), it is likely that AI articles have been published with various search terms or in different specialty journals that could explain why the initial review did not capture these additional eight agents. Nevertheless, the inclusion of these additional eight agents allows for a more holistic view of a MASH framework. Moreover, our review primarily focused on elective perioperative care; AI applications in emergency or trauma surgery were not systematically included, which may limit generalizability to acute settings. Future reviews should incorporate emergency contexts to fully capture the continuum of perioperative AI utility.

### 4.1. Opportunities for Anesthesiology

The perioperative environment is uniquely suited to benefit from AI integration. Predictive tools provide early warning signs of physiologic deterioration, enabling preemptive intervention and thus enhanced patient safety. AI could also assist with practical tasks, thereby improving economic efficiency, as automated documentation and optimized scheduling could reduce administrative burden and resource waste. Most importantly, integrated systems in machine learning and the feedback loops and communication embedded within MASH allow for tailoring of anesthetic, ventilatory, and analgesic strategies that can refine performance within and across patient encounters.

### 4.2. Implementation Challenges

Several barriers must be addressed before widespread adoption, as most perioperative AI tools are not interoperable across various electronic health record (EHR) systems. Development of a clinically useful MASH network will entail integration within broader EHR infrastructures. It will also necessitate a collaborative team of AI specialists, clinicians, and informaticians to ensure that implementation adheres to technical and regulatory standards.

First, there is the need to establish a central “orchestrator” agent that acts to direct tasks to specialized AI agents that each handle specific clinical functions (documentation, decision support, analysis, etc.). Second, any MASH system must connect these agents through secure communication methods using standardized healthcare interoperability protocols such as the Fast Healthcare Interoperability Resources developed by Health Level Seven International (FHIR/HL7) [34]. These frameworks enable structured data exchange across heterogeneous HER platforms while maintaining compliance with patient privacy regulations such as the Health Insurance Portability and Accountability Act (HIPAA) and the General Data Protection Regulation (GDPR). Achieving interoperability globally remains challenging, as institutions will differ in data architecture, regulatory standards, as well as their willingness to share information. Thus, successful MASH usage will depend on establishing common data standards, transparent consent mechanisms, and streamlined institutional policies that facilitate data access.

Finally, when viewed through a cybersecurity lens, distributed systems and architectures inherently expand the potential for software and technological attacks. The more connections between agents, the greater the potential for unauthorized access or data leakage. Potential strategies to mitigate this risk should include encryption of data in transit and at baseline, regular vulnerability assessments, and implementation of role-based or task-specific permissions, ensuring that each agent only accesses the minimum necessary data.

Medicolegal and ethical considerations are also important. Automated decision support in anesthesiology raises questions regarding the liability in cases of adverse outcomes—would responsibility lie with the clinician, institution, or system developer? Legal frameworks will also need to evolve to address shared accountability models, and professional/society guidelines should emphasize that AI recommendations would augment, never replace, anesthesiologists’ judgment. Alarm-fatigue, equivocal decision-making, and poor sensitivity and/or specificity could erode trust if not carefully managed.

Above all, AI implementation in perioperative care must comply with existing frameworks for patient safety, accountability, and privacy, while addressing emerging concerns about algorithmic transparency and interoperability across diverse healthcare systems.

### 4.3. Future Directions and Implications

Currently, clinical evidence supporting large-scale implementation remains limited. Most perioperative AI studies are proof-of-concept or retrospective validations rather than prospective, real-world evaluations. Moving toward routine implementation will require multicenter trials designed to assess safety, performance, and equity, along with robust governance frameworks to ensure ethical oversight.

Early implementation of AI in anesthesiology should focus on low-risk, high-verifiability tasks, such as documentation support or predictive scheduling, before progressing to high-acuity applications like autonomous intraoperative decision support. Prospective validation through multicenter trials will be essential to establish generalizability and safety, with careful attention to study design to ensure equity and mitigate bias. Importantly, future frameworks must include interdisciplinary collaboration among anesthesiologists, data scientists, and ethicists in co-designing AI systems to ensure clinical relevance and safety.

## 5. Conclusions

Anesthesiologists are uniquely positioned to lead AI integration because of their central role in perioperative care, critical care, and pain management. By adopting a MASH approach, perioperative teams can transform AI from fragmented, task-specific tools into an interconnected support network that adapts to evolving patient needs. Rather than replacing clinical judgment, these systems can strengthen communication during transitions of care, provide real-time decision support, and drive continuous quality improvement. Ultimately, coordinated AI integration has the potential to make perioperative care more personalized, more efficient, and most importantly, safer.

## Data Availability

No new data were created or analyzed in this study. Data sharing is not applicable to this article.
